# Creating Clinical Reasoning Assessment Tools in Different Languages: Adaptation of the Pediatric Emergency Medicine Script Concordance Test to Japanese

**DOI:** 10.3389/fmed.2021.765489

**Published:** 2021-12-07

**Authors:** Osamu Nomura, Taichi Itoh, Takaaki Mori, Takateru Ihara, Satoshi Tsuji, Nobuaki Inoue, Benoit Carrière

**Affiliations:** ^1^Department of Emergency and Disaster Medicine, Hirosaki University, Hirosaki, Aomori, Japan; ^2^Department of Emergency Medicine, University of Michigan, Ann Arbor, MI, United States; ^3^Department of Medical Education, University of Illinois at Chicago, Chicago, IL, United States; ^4^Department of Emergency Medicine, KK Women's and Children's Hospital, Singapore, Singapore; ^5^Division of Pediatric Emergency Medicine, Tokyo Children's Medical Center, Fuchu, Tokyo, Japan; ^6^National Center for Child Health and Development, Setagaya, Tokyo, Japan; ^7^Department of Human Resources Development, Bureau of International Health Cooperation, National Center for Global Health and Medicine, Shinjuku, Tokyo, Japan; ^8^Department of Pediatrics, Section of Pediatric Emergency Medicine, Centre Hospitalier Universitaire Sainte-Justine, Montréal, QC, Canada

**Keywords:** clinical reasoning assessment, Script Concordance Test, pediatric emergency medicine education, competency, validating a measure

## Abstract

**Introduction:** Clinical reasoning is a crucial skill in the practice of pediatric emergency medicine and a vital element of the various competencies achieved during the clinical training of resident doctors. Pediatric emergency physicians are often required to stabilize patients and make correct diagnoses with limited clinical information, time and resources. The Pediatric Emergency Medicine Script Concordance Test (PEM-SCT) has been developed specifically for assessing physician's reasoning skills in the context of the uncertainties in pediatric emergency practice. In this study, we developed the Japanese version of the PEM-SCT (Jpem-SCT) and confirmed its validity by collecting relevant evidence.

**Methods:** The Jpem-SCT was developed by translating the PEM-SCT into Japanese using the Translation, Review, Adjudication, Pretest, Documentation team translation model, which follows cross-cultural survey guidelines for proper translation and cross-cultural and linguistic equivalences between the English and Japanese version of the survey. First, 15 experienced pediatricians participated in the pre-test session, serving as a reference panel for modifying the test descriptions, incorporating Japanese context, and establishing the basis for the scoring process. Then, a 1-h test containing 60 questions was administered to 75 trainees from three academic institutions. Following data collection, we calculated the item-total correlations of the scores to optimize selection of the best items in the final version of the Jpem-SCT. The reliability of the finalized Jpem-SCT was calculated using Cronbach's α coefficient for ensuring generalizability of the evidence. We also conducted multiple regression analysis of the test score to collect evidence on validity of the extrapolation.

**Results:** The final version of the test, based on item-total correlation data analysis, contained 45 questions. The participant's specialties were as follows: Transitional interns 12.0%, pediatric residents 56.0%, emergency medicine residents 25.3%, and PEM fellows 6.7%. The mean score of the final version of the Jpem-SCT was 68.6 (SD 9.8). The reliability of the optimized test (Cronbach's α) was 0.70. Multiple regression analysis showed that being a transitional intern was a negative predictor of test scores, indicating that clinical experience relates to performance on the Jpem-SCT.

**Conclusion:** This pediatric emergency medicine Script Concordance Test was reliable and valid for assessing the development of clinical reasoning by trainee doctors during residency training.

## Introduction

Clinical reasoning is central to being a health professional (1), such skills being even more critical in the emergency department, where multiple patients are treated simultaneously (2, 3). Pediatric emergency physicians are often required to stabilize patients and make correct diagnoses with limited clinical information, time and resources. This requires not only deep understanding and knowledge of the pathophysiology and presentation of a wide variety of illnesses, but also well-developed abilities for clinical reasoning. From a health profession education perspective, assessing the competency of clinical reasoning in emergency medicine is critically important to assure medical trainee's competency, and hence, the quality of care in the emergency department (4). The script concordance test (SCT) has been utilized to assess the clinical reasoning competency of physicians interpreting medical information under such uncertain conditions, as in the emergency department. The SCT was introduced by Charlin and collaborators based on the Script theory, which postulates that in specific situations, clinicians mobilize pre-stored sets of knowledge, i.e., scripts, that are used to understand the situation and act according to specific goals, whether diagnostic, investigative or therapeutic (5). The test is a written simulation exercise designed to assess the ability to weigh information in light of possible hypotheses in ill-defined clinical situations. The test approach consists of presenting examinees with a series of patient vignettes and then asking them to make diagnostic, investigative, or therapeutic decisions based on specific elements of the information provided. The test is designed to probe how the decisions made based on a clinical reasoning process are similar to the decisions made by a reference panel of experts.

The principle behind the SCT is to compare the scripts of examinees with those of experienced clinicians using a series of clinical tasks set in specific contexts (6, 7). The scoring system is designed to measure the concordance between examinee's scripts and scripts of a panel of experts. Diagnostic hypotheses, investigative strategies and treatment options are specified for each situation. Short clinical vignettes, each followed by a series of test questions, make up the SCT. Each case vignette is followed by three parts. The first part (“If you were thinking of”) contains a diagnosis, investigation, or treatment relevant to the clinical vignette. The second part (“And then you find”) presents information, such as a physical sign, a pre-existing condition, an imaging study, or a laboratory test result that might affect the first part. The third part (“This hypothesis becomes”) is a five-point Likert scale that the examinee uses to indicate what effect this information (part 2) has on the proposed diagnosis, investigation, or treatment (part 1). This scoring method is termed aggregate scoring, and was initially proposed by Norman, and later Norcini, (as described by Lubarsky et al. and Dory et al.) (8, 9). In this scoring system, the most plausible answer selected by the largest number of expert panelists (i.e., the modal answer) is considered the “gold standard” reasoning under the given circumstances, and the number of panelists who select the modal answer is known as the modal value.

When scoring each question, examinee's answers receive a credit mark corresponding to the proportion of panel members who selected that particular answer on the scale. The maximum score for each question is one (1) for the modal answer. Other panel member's choices receive a partial credit. Answers not chosen by panel members receive a score of zero. To obtain proportional transformation, the number of members who selected a particular answer on the Likert scale is divided by the modal value (i.e., the number of expert panelists who selected the modal answer) for the item. For example, if there are 15 members in the reference panel who answered a question on a given SCT in the following way: none chose the “−2” and “−1” ratings, two chose the “0” rating, nine answered the “+1” rating, and four chose the “+2” rating, the modal answer in this example would be the “+1” rating and the modal value would be nine. Choosing this rating will give the examinee 1.0 point for that response. Those who select “0” rating as their response will receive 0.22 points (2/9) and examinees who select “+2” rating as their answer will receive 0.44 points (4/9).

Although much research has been performed on the effectiveness of the SCT in assessing clinical reasoning ability, there has been little research on the SCT in Japan. In the context of pediatric emergency medicine, the Pediatric Emergency Medicine Script Concordance Test (PEM-SCT) was developed in Canada as a tool for assessing clinical reasoning competency in pediatric emergency medicine (Supplementary Appendix 1), and was shown to be highly reliable and valid (10). The purpose of this study was to develop and validate a Japanese version of the PEM-SCT (Jpem-SCT), to measure the clinical reasoning ability in pediatric emergency medicine of Japanese medical trainees. Our study also describes the methodology of developing versions of the SCT in any language by translating the English SCT into the required language.

This study was conducted in two phases: (1) development of the Jpem-SCT and (2) validation of the Jpem-SCT, with 15 supervising physicians and 75 post-graduate trainee doctors in charge of pediatric emergency care at three tertiary facilities as participants.

## Methods

### Development of the Jpem-SCT

**Figure 1:**
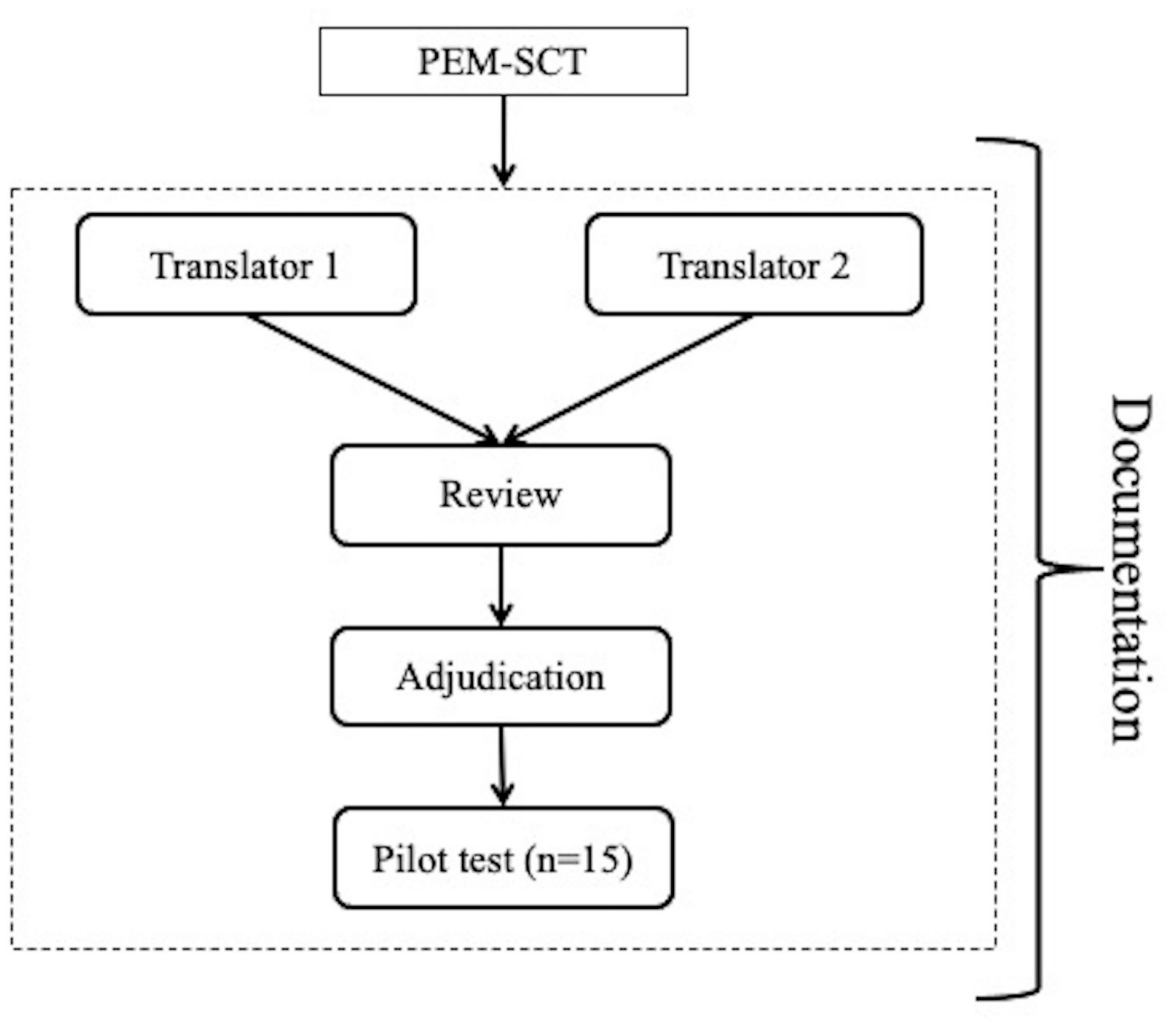
Translation, review, adjudication, pretest, documentation team model for the development of the Japanese version of the pediatric emergency medicine script concordance test.

The Jpem-SCT was developed by translating the PEM-SCT into Japanese using the Translation, Review, Adjudication, Pretest, Documentation (TRAPD) team translation model (11), which follows cross-cultural survey guidelines to ensure proper translation and cross-cultural and linguistic equivalence between the English and Japanese survey versions. We formed a bilingual translation committee consisting of translators, a translation reviewer, and an adjudicator (Figure 1). First, two pediatric emergency physicians with experience in pediatric emergency practice in Japan and English-speaking countries (i.e., the United States and Singapore) individually translated the entire PEM-SCT into Japanese. Then, one reviewer, a pediatric emergency physician trained in Japan and the United States and who has experience translating English books on pediatric emergency medicine into Japanese, examined all the items and identified those with potential problems from a pediatric emergency medicine and linguistic perspective. The principal investigator, a pediatric emergency physician with a Master's degree in Medical Education, adjudicated the final version of the Jpem-SCT with the agreement of the translators and reviewer. As the final step of the TRAPD team translation model, the draft version was evaluated for comprehensibility, a Jpem-SCT scoring rubric was created by the expert panel, and a pre-test was conducted. In the pre-test, 15 non-bilingual pediatric emergency physicians at participating facilities were asked to complete the draft Jpem-SCT and their response data was collected. The scoring rubric was created using a scoring tool developed and published by the University of Montreal (12).

The committee convened two three-hour meetings to discuss all the items and any possible issues based on the pre-test responses, and revised the descriptions of antibiotics and transfusion therapy to correspond to the Japanese medical situation. The Jpem-SCT was appropriately modified based on the discussions and feedback from the meetings. The modified version of the Jpem-SCT contained a total of 60 questions on 38 cases and consisted of questions on diagnosis, laboratory procedures and treatment.

### Validation of the Jpem-SCT

A total of 75 post-graduate trainee doctors, including transitional interns (PGY1 and 2), pediatric residents (PGY3-5), emergency medicine residents (PGY3-5), and pediatric emergency medicine fellows at the three participating institutions were invited to participate in a 60-min session of the Jpem-SCT (60 questions on 38 cases). The sample size was determined by adjusting the statistical power of the original PEM-SCT study (10). Using three visual analog scales, the examinees also responded to a questionnaire on time allotted to complete the task, the similarity of the cases compared to real-life clinical problems, and the perceived level of difficulty of the Jpem-SCT. The data collection sessions were held *via* a video-conference system due to the prevailing COVID-19 pandemic, and the trainees joined the sessions from their workplace or home. After the data collection, the participant's responses were entered into the scoring tool to calculate the Jpem-SCT scores (12).

### Optimization of the Jpem-SCT

In striving to obtain the best possible discrimination for the Jpem-SCT, an item-total correlation test was performed, followed by an iterative process for eliminating items with low correlation coefficients (*r* ≤ 0.1). This analysis produced optimized scores. Post-optimization reliability coefficients (Cronbach's α) were obtained (2, 4, 8). Multiple regression analysis was conducted with the Jpem-SCT score as the dependent variable and the participant's demographic data as the independent variable to develop a validity argument.

### Validation Framework

Traditionally, the process of validation involves analyzing several types of validity, such as face validity, content validity, convergent validity and criterion validity. Messick suggested abandoning these types of validity and instead, unifying them under “construct validity,” and proposed a framework with five evidence sources for validation, including content, response process, internal structure, relation to other variables, and consequences (13). However, Messick's framework does not prioritize these sources of validity evidence. Kane's adaptation of Messick's framework addresses this shortcoming by ordering and linking each of the steps in collecting validity information, to create a visible and logical chain of evidence that exhibits the strengths and weaknesses of each inference within a validity argument (14). Kane identified four key elements for inference of validity arguments to help visualize the steps in validation: (a) scoring of a single observation (Scoring); (b) using the primary observation score data to generate a picture of overall test performance (Generalization); (c) inferring real-life performance from test performance (Extrapolation); and (d) interpreting this information and making a decision (Implication). Kane emphasized implication as the most important step in the validity argument in real-world settings. Based on the obtained data, we examined validity evidence for the Jpem-SCT in terms of Kane's validity arguments (14), as this framework is useful for validating translated assessment tools (15).

### Ethical Aspects

This study was approved by the ethics committees of the three participating institutions: Hirosaki University (2020–029), the National Center for Child Health and Development, Tokyo (2020–079), and Tokyo Metropolitan Children's Medical Center (2020b−29).

## Results

The participants included 9 transitional interns (PGY1 and 2), 41 pediatric residents (PGY3-5), 18 emergency medicine residents (PGY3-5), and seven pediatric emergency medicine fellows, with an average of 4.7 years of post-graduate training. Fifteen questions were eliminated by the optimization process to remove items with low correlation coefficients (*r* ≤ 0.1) in the item-test correlation test, and the final version of the Jpem-SCT consisting of 45 questions was created. The score was converted to a percentage (i.e., a perfect score on all 45 questions meant 100%), which was automatically calculated by the scoring tool of the University of Montreal (12). The mean score of the 75 trainee doctors was 68.6% (SD 9.8). Cronbach's α for evaluation of the reliability of the optimized test was 0.70 (indicating generally high reliability).

The mean level of difficulty of the JPEM-SCT as perceived by the participants was 6.6 (10: most difficult, SD 1.4). For the question regarding the time required to complete the responses (with 1 being “there was enough time” and 10 being “not enough time”), the mean response was 1.4 (SD 1.7); the rating for whether the content of the Jpem-SCT represented actual clinical situations was a mean of 6.7 (10 being the best, SD 1.8) (Table 1). The results of multivariate analysis of Jpem-SCT scores showed that being a transitional intern was a negative predictor of a good test score (β = −*0.26, p* = 0.04) (Table 2).

**Table 1:** Demographics of the participants.

**Variables**	
Postgraduate years (years), Mean (SD)	4.7 (2.4)
Training specialty, *n* (%)	
Transitional interns	9 (12.0)
Pediatric resident	41 (54.7)
Emergency medicine resident	18 (24.0)
Pediatric emergency medicine fellow	7 (9.3)
Perceived level of difficulty (score: 0–10), Mean (SD)	6.6 (1.4)
Time allotted to complete the task (score: 0–10), Mean (SD)	1.4 (1.7)
Similarity to real-life clinical problems (score: 0–10), Mean (SD)	6.7 (1.8)
Jpem-SCT score (0–100), Mean (SD)	68.6 (9.8)

*SD, standard deviation; Jpem-SCT, Japanese version of the Pediatric Emergency Medicine Script Concordance Test*.

**Table 2:** Multiple regression analysis of the Japanese version of the pediatric emergency medicine script concordance test score.

**Variables**	**Adjusted β**	**95% CI**	***p*-value**
Constant		56.0 to 80.5	<0.01
Postgraduate year	0.21	−0.26 to 1.93	0.13
Training specialty, *n* (%)	
Pediatric resident (Reference)	–	–	–
Transitional intern	−0.26	−15.29 to 0.45	0.04
Emergency medicine resident	−0.05	−6.42 to 4.31	0.7
Pediatric emergency medicine fellow	0.12	−4.16 to 12.4	0.32
Perceived level of difficulty	−0.06	−2.02 to 1.14	0.58
Time allotted to complete the task	0.18	−1.25 to 1.46	0.88

*R^2^ = 0.221 (p < 0.05)*.

## Discussion

We developed the Jpem-SCT, the Japanese version of a clinical reasoning skills assessment tool specific to pediatric emergency medicine. We also examined validity evidence for the Jpem-SCT in terms of the four elements of Kane's validity arguments, namely, (1) scoring, (2) generalization, (3) extrapolation, and (4) implications (14), and discuss this evidence below. This study also presents a summary of the methodology for successfully translating the original English version of the SCT to another language, and for validating the translated SCT, which will promote clinical reasoning research in various contexts.

### Inference Regarding Scoring

Our study indicated the appropriateness of collecting data and scoring clinical reasoning ability using the Jpem-SCT. The pre-test with the draft Jpem-SCT was designed to allow respondents to fully understand the hypothetical clinical vignettes, which was achieved by asking a panel of experts to comment on the appropriateness of the language in the statements and asking them to revise them as required. Evaluation of the test results and the participants rating of the test indicated that they were able to complete the modified version of the Jpem-SCT in a timely manner and there were no questions that could not be understood. In addition, the score for perceived test difficulty was similar to those in previous studies. While data on individual perceptions is less objective than quantitative data, the examinee's perceptions are essential for inferring the scoring phase in Kane's framework (15). Our inferences revealed that the experts and trainees evaluated the Jpem-SCT as having high clarity and usability, indicating that the TRAPD team translation model was effective.

### Inference Regarding Generalizability

To strengthen the generalizability of the test, the Jpem-SCT was optimized by calculating item-test correlations for the scores of 60 responses, and deleting inappropriate questions to create a generalizable 45-question Jpem-SCT. The reliability coefficient (Cronbach's α) of the final version of the Jpem-SCT, finalized through the optimization process, was generally high (0.70), suggesting that it has adequate psychometric evidence for generalization.

### Inference Regarding Extrapolation

The Jpem-SCT is designed to assess clinical reasoning skills among pediatric emergency medicine specialists, and its target examinees are pediatric and emergency medicine residents and pediatric emergency medicine fellows in Japan. In this context, the fact that being a transitional intern (PGY1 and 2) was a negative predictor of a good score (i.e., the questions were too difficult for early trainees) indicates the positive association between the subject's clinical reasoning ability (acquired through training and experience) and the Jpem-SCT score (16). This finding provides reasonable evidence on the ease of extrapolation of the Jpem-SCT to other assessment settings.

### Inference Regarding Implementation

The implementation inference argues the applicability of the Jpem-SCT score to clinical education. The participants responded that the Jpem-SCT effectively represents actual clinical situations (mean value: 6.7). We, therefore, confirmed that the Jpem-SCT could be utilized for clinical education in the field of pediatric emergency medicine in Japan.

### Strengths and Limitations

To the best of our knowledge, this is the first study validating the SCT in the East Asian medical education context. Sociocultural factors, such as parenting culture and the healthcare system, often influence pediatric emergency practice in each country, and differences in cultural background can be a barrier to the accurate translation of assessment tools in medical education. To deal with this issue, we utilized the TRAPD team model, employing bilingual experts to aim for cross-cultural concordance and equivalence between English and Japanese versions of the PEM-SCT, which aided successful development of the Jpem-SCT for accurate assessment of trainee's competency in pediatric emergency medicine. This might expand the possibilities for conducting cross-country research, comparing clinical reasoning competency of pediatric emergency medicine trainees across multiple countries(17).

There are a few limitations to this study. The proportion of participant's of each specialty was uneven as this study was conducted during the year of the COVID-19 pandemic; hence, it was difficult to invite emergency medicine residents to this study (18). However, the performance of emergency medicine resident's in terms of Jpem-SCT scores did not differ from those of pediatric residents; thus, the influence of the uneven proportion of participants from each specialty on the study results might be minimal.

## Conclusion

We developed and validated a Japanese version of the SCT specifically for use in the field of pediatric emergency medicine, and confirmed that it could be used for assessment of clinical education about pediatric emergencies in Japan.

## Data Availability Statement

The raw data supporting the conclusions of this article will be made available by the authors, without undue reservation.

## Ethics Statement

The studies involving human participants were reviewed this study was approved by the Ethics Committees of the three participating institutions: Hirosaki University (2020–029), the National Center for Child Health and Development, Tokyo (2020–079), and Tokyo Metropolitan Children's Medical Center (2020b−29). The participants consented to participate in this study by filling out the electronic consent form.

## Author Contributions

ON: designed this study, collected data, performed the statistical analyses, and drafted the manuscript. TIt, TM, and NI: supported the development of the measurement tool. TIh and ST: supported data collection. BC: reviewed the manuscript. All authors read and approved the final manuscript.

## Funding

This study was supported by a grant from the Japan Medical Education Foundation and the Japan Foundation for Pediatric Research. The APC was funded by Tohoku Initiative Fostering Global Researchers for Interdisciplinary Sciences.

## Conflict of Interest

The authors declare that the research was conducted in the absence of any commercial or financial relationships that could be construed as a potential conflict of interest.

## Publisher's Note

All claims expressed in this article are solely those of the authors and do not necessarily represent those of their affiliated organizations, or those of the publisher, the editors and the reviewers. Any product that may be evaluated in this article, or claim that may be made by its manufacturer, is not guaranteed or endorsed by the publisher.

## Abbreviations

SCT: Script Concordance Test
PEM-SCT: Pediatric Emergency Medicine Script Concordance Test
Jpem-SCT: Japanese version of the Pediatric Emergency Medicine Script Concordance Test
TRAPD, Translation, Review, Adjudication, Pretest: Documentation.
