# Supplementary material for: Creating Clinical Reasoning Assessment Tools in Different Languages: Adaptation of the Pediatric Emergency Medicine Script Concordance Test to Japanese
Source: Front Med (Lausanne). 2021 Dec 7;8:765489. doi: 10.3389/fmed.2021.765489 (PMC8688734; doi:10.3389/fmed.2021.765489)
Supplement: Supplementary file 1 [file Table_1.DOCX]

Appendix 1

The Excel corrector program for Script Concordance Tests (SCTs) is downloadable from this link.

http://wp-portail.med.umontreal.ca/cpass/wp-content/uploads/sites/4/2015/07/TCS-2011-.xlsm
