# Supplementary material for: Creating Clinical Reasoning Assessment Tools in Different Languages: Adaptation of the Pediatric Emergency Medicine Script Concordance Test to Japanese
Source: Front Med (Lausanne). 2021 Dec 7;8:765489. doi: 10.3389/fmed.2021.765489 (PMC8688734; doi:10.3389/fmed.2021.765489)
Supplement: Supplementary file 2 [file Data_Sheet_1.pdf]

Supplementary Appendix 1. Example of the Pediatric Emergency Medicine Script Concordance Test case: A 3-year-old girl arrives with major ptyalism, a decrease in the mobility of her cervical spine, and a fever that has lasted more than 24 hours. Her parents do not mention any history of trauma or obstruction by foreign matter.

**If you have been considering:**	**And you then discover**	**The effect on your diagnostic hypothesis is the following**
Epiglottitis	An up-to-date Haemophilus influenzae B vaccination	**-2 -1 0 +1 +2**
A retropharyngeal abscess	A negative throat culture for Streptococcus A	**-2 -1 0 +1 +2**

-2=Ruled out or almost ruled out; -1=less probable; 0=neither less nor more probable; +1=more probable; +2=certain or almost certain
